# Characteristics of Patients Lost to Follow-up after Bariatric Surgery

**DOI:** 10.3390/nu16162710

**Published:** 2024-08-15

**Authors:** Laura Krietenstein, Ann-Cathrin Koschker, Alexander Dimitri Miras, Lars Kollmann, Maximilian Gruber, Ulrich Dischinger, Imme Haubitz, Martin Fassnacht, Bodo Warrings, Florian Seyfried

**Affiliations:** 1 Department of General, Visceral, Transplantation, Vascular and Paediatric Surgery, University Hospital Würzburg, 97080 Würzburg, Germanykollmann_l@ukw.de (L.K.); gruber_m@ukw.de (M.G.);; 2 Department of Internal Medicine I, Division of Endocrinology and Diabetology, University Hospital Würzburg, 97080 Würzburg, Germany; koschker_a@ukw.de (A.-C.K.); dischinger_u@ukw.de (U.D.); fassnacht_m@ukw.de (M.F.); 3 School of Medicine, Ulster University, Derry BT47 6AE, UK; a.miras@nhs.net; 4 Department of Psychiatry, Psychosomatics, and Psychotherapy, Centre for Mental Health, University Hospital Würzburg, 97080 Würzburg, Germany; warrings_b@ukw.de

**Keywords:** bariatric surgery, metabolic surgery, follow-up, aftercare, lost to follow-up, micronutrient supplementation

## Abstract

After bariatric surgery lifelong follow-up is recommended. Evidence of the consequences and reasons for being lost to follow-up (LTFU) is sparse. In this prospective study follow-up data of all patients who underwent bariatric surgery between 2008 and 2017 at a certified obesity centre were investigated. LTFU patients were evaluated through a structured telephone interview. Overall, 573 patients (female/male 70.9%/29.1%), aged 44.1 ± 11.2 years, preoperative BMI 52.1 ± 8.4 kg/m^2^ underwent bariatric surgery. Out of these, 33.2% had type 2 diabetes mellitus and 74.4% had arterial hypertension. A total of 290 patients were LTFU, of those 82.1% could be reached. Baseline characteristics of patients in follow-up (IFU) and LTFU were comparable, but men were more often LTFU (*p* = 0.01). Reported postoperative total weight loss (%TWL) and improvements of comorbidities were comparable, but %TWL was higher in patients remaining in follow-up for at least 2 years (*p* = 0.013). Travel issues were mentioned as the main reason for being LTFU. A percentage of 77.6% of patients reported to regularly supplement micronutrients, while 71.0% stated regular monitoring of their micronutrient status, mostly by primary care physicians. Despite comparable reported outcomes of LTFU to IFU patients, the duration of the in-centre follow-up period affected %TWL. There is a lack of sufficient supplementation and monitoring of micronutrients in a considerable number of LTFU patients.

## 1. Introduction

Metabolic or bariatric surgery is currently the most effective treatment for obesity WHO III°. It does not only facilitate long-term weight loss but improves obesity-related comorbidities [1,2,3] and extends life expectancy [4].

After metabolic or bariatric surgery, lifelong follow-up is recommended according to national and international guidelines [5,6]. Key components of the follow-up routine include regular nutritional counselling, laboratory tests to detect possible micronutrient deficiencies, monitoring of body weight loss, adjusting medication for comorbidities and encouraging patients to stay physically active and to participate in support groups. Additionally, a screening for mental health disorders should be conducted. Routine follow-up is recommended after one, three, six, twelve and eighteen months and then annually after surgery [5].

Currently, standardised follow-up of patients undergoing bariatric surgery in Germany is almost exclusively carried out by the centre where they have been operated, as there is no reimbursement for the follow-up to take place elsewhere [7,8,9]. Considering that the total number of patients who have undergone surgery is increasing and the necessity of postoperative lifelong follow-up, the already limited capacity of these specialist centres will become critical [10,11].

Existing data indicate that the majority of patients become “lost to follow-up” (LTFU) over time [12,13]. Adherence to follow-up recommendations is generally reported to be about 90% in the first year and then decreases drastically to reach 30% in two years and less than 10% in 10 years [14]. Since LTFU patients often consult their obesity centre when they experience major difficulties, it is generally assumed that being LTFU is associated with inferior postoperative outcomes [15,16].

However, the existing literature on the consequences of being LTFU is sparse, often covering limited time periods and showing inconsistent results for clinically relevant parameters [17,18,19]. Even less is known about the reasons why patients drop out of the recommended routine follow-up. Additionally, it is unclear whether these data can be transferred to a German bariatric patient collective, which is known to be older, sicker, and with more obesity at the time of surgery compared to other countries around the world [20].

It is therefore important to examine the postoperative outcomes of LTFU patients and to review the reasons for their drop-out from follow-up more closely.

In this study we aim to explore (i) whether there are differences in preoperative characteristics and postoperative outcomes between IFU and LTFU patients, (ii) whether the duration of participation in follow-up affects long-term outcomes, (iii) who monitors a patient’s health status and ensures the necessary supplementation of micronutrients of patients LTFU and (iv) what the reasons are for patients to drop out of routine follow-up after bariatric surgery.

## 2. Materials and Methods

This is a prospective single centre study investigating data from all consecutive patients who underwent bariatric surgery according to the evidence-based national S3 guidelines [5] at the certified obesity centre, University Hospital of Würzburg, Germany, between September 2008 and October 2017. This time frame was used to have a sufficient long follow-up time for all patients. Patient data were prospectively collected and entered into the prospective national registry of the German Society for General and Visceral Surgery (DGAV) StuDoQ|MBE following patients’ informed consent [21,22,23].

The analysis of the prospective StuDoQ|MBE database included patients’ baseline characteristics (age, sex, body weight, comorbidities, current medication and HbA1c) as well as postoperative complications, reoperations, changes in body weight, comorbidities and medications. Follow-up data were taken from patients’ records as long as they were in follow-up.

Patients were classified according to their follow-up status. Patients who had not attended a follow-up appointment within the previous 18 months were classified as LTFU. Data of LTFU were collected through a structured telephone interview (patient reported outcomes, PROs). All patients were informed about the collection of anonymised data for study purposes at the beginning of the interview; verbal consent was obtained and documented. Participation could be declined or the interview terminated at any time. Patients were contacted between July 2019 and August 2019. A maximum of five call attempts at different daytimes and days were made. If patients were still ‘not available’, they were recorded as such accordingly. If patients declined the interview, they were noted as ‘available, but no interest’. Patients who participated in the telephone interview are referred to as ‘questioned LTFU’ (qLTFU). The study was approved by the local ethics committee (2019-0430 01).

Patients’ baseline characteristics (age, sex, body weight, comorbidities, medications and type of bariatric procedure) as well as postoperative changes in body weight, comorbidities and medications over time were compared between IFU and LTFU patients. Additionally, LTFU patients were stratified according to the time they had taken part in the routine follow-up at the centre before LTFU (<2 years and ≥2 years) for further subgroup analysis. Deceased patients were excluded in this context, but descriptive data are shown.

The structured telephone interview of LTFU patients focused on patients’ satisfaction, postoperative issues, mental well-being, reasons for being LTFU, supplementation of micronutrients and further care.

Statistical analyses were performed using MEDAS software by Grund, Margetshöchheim, version 5/23. Statistical analyses included the Chi-square test by the maximum likelihood method (p_c_), the exact Chi-square test by Fisher and Yates (p_fy_) for smaller samples, and the exact Chi-square test by Mehta and Patel (p_mp_) for more than two groups to describe relationships between two variables. The independent t-test (p_t_) was used to check for differences in mean values. To determine if a Gaussian normal distribution was present, the probit plot with Lilliefors’ bounds was used. If a Gaussian normal distribution could not be assumed, the Mann–Whitney U test (p_U_) was used to check the equality of distribution between two samples. For comparing more than two groups (and if Gaussian normal distribution was not assumed), the Kruskal–Wallis one-way analysis of variance was used to test for equality of means across different groups. If a Gaussian normal distribution could be assumed, the one-way ANOVA (p_a_) was used. Correlations were calculated using Pearson’s correlation coefficient (r) if a Gaussian normal distribution was present. If not, Kendall’s rank correlation coefficient (τ) was calculated. *p*-values of <0.05 were considered statistically significant. Descriptive statistics included mean, minimum and maximum values and standard deviation.

Statistical analyses were performed using MEDAS software by Grund, Margetshöchheim, version 5/23. Statistical analyses included the Chi-square test by the maximum likelihood method (p_c_), the exact Chi-square test by Fisher and Yates (p_fy_) for smaller samples, and the exact Chi-square test by Mehta and Patel (p_mp_) for more than two groups to describe relationships between two variables. The independent t-test (p_t_) was used to check for differences in mean values. To determine if a Gaussian normal distribution was present, the probit plot with Lilliefors’ bounds was used. If a Gaussian normal distribution could not be assumed, the Mann–Whitney U test (p_U_) was used to check the equality of distribution between two samples. For comparing more than two groups (and if Gaussian normal distribution was not assumed), the Kruskal-Wallis one-way analysis of variance was used to test for equality of means across different groups. If a Gaussian normal distribution could be assumed, the one-way ANOVA (p_a_) was used. Correlations were calculated using Pearson’s correlation coefficient (r) if a Gaussian normal distribution was present. If not, Kendall’s rank correlation coefficient (τ) was calculated. *p*-values of <0.05 were considered statistically significant. Descriptive statistics included mean, minimum and maximum values, and standard deviation.

## 3. Results

From September 2008 to October 2017, 591 patients underwent metabolic or bariatric surgery, receiving either laparoscopic sleeve gastrectomy (SG), Roux-en-Y gastric bypass (RYGB), or conversion from an SG to an RYGB at the University Hospital of Würzburg.

**Figure 1:**
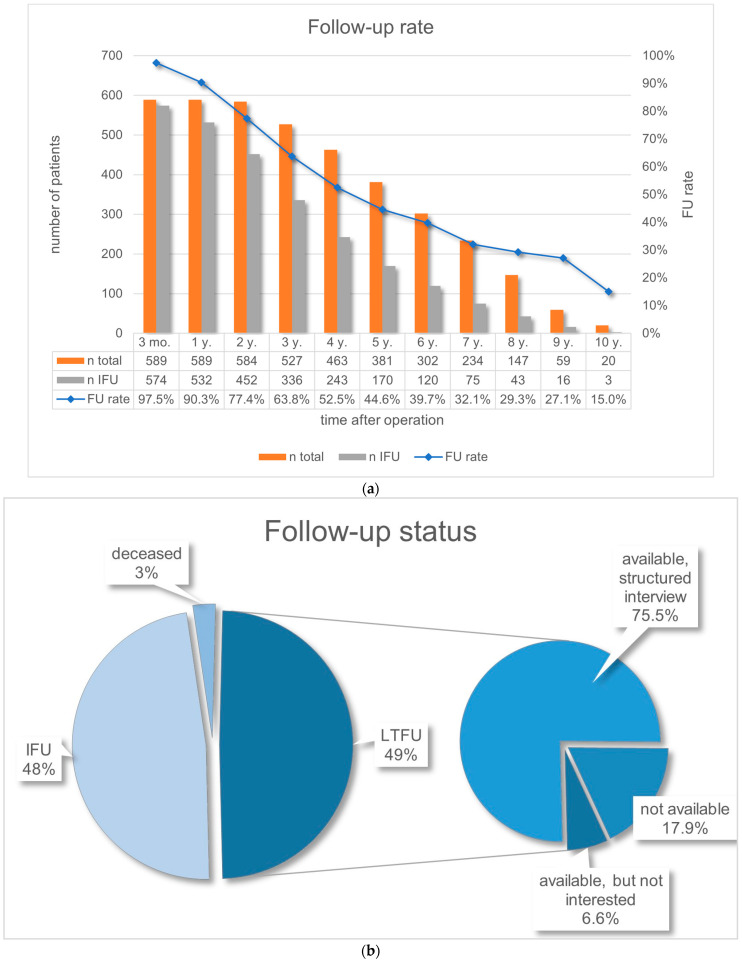
(**a**) Follow-up rate; IFU = in follow-up; y. = year; mo. = months; n = number. (**b**) Follow-up status; IFU = in follow-up; LTFU = lost to follow-up.

The first-year follow-up rate was 90.32%, continuously decreasing thereafter (Figure 1a). The proportion of attended appointments over the entire period in the IFU group was 83.92%.

According to patients’ records, 16 patients had died, eight of whom met the criteria for LTFU. Overall, 283 patients were IFU and 290 patients were LTFU. On average, the LTFU patients had been in follow-up for 2.2 years after surgery and the operation was 7.0 years ago. Of 290 LTFU patients, telephone interviews were conducted with 219 (72.1%). Nineteen patients (6.6%) expressed no interest (‘available, but no interest’), and 52 (17.9%) patients could not be reached (Figure 1b), resulting in a total follow-up rate of 87.6% as of July 2019.

### 3.1. Baseline Characteristics of IFU vs. LTFU Patients

Baseline characteristics of IFU and LTFU patients are presented in Table 1. The preoperative BMI was 52.1 ± 8.4 kg/m^2^, the average age at the time of surgery was 44.1 ± 11.2 years. Type 2 diabetes mellitus had been diagnosed in 33.2% (*n* = 176) of patients preoperatively. Of these, 9.7% (*n* = 17) of cases were diet-controlled, 53.4% (*n* = 94) received non-insulin antidiabetic drugs (NIAD), and 36.9% (*n* = 65) insulin treatment. Arterial hypertension was diagnosed in 74.4% (*n* = 395) of the patients prior to surgery. Of these, 19.0% (*n* = 67) received no therapy, 28.9% (*n* = 102) received single drug therapy and 52.1% (*n* = 184) multiple drug therapy. Depression was diagnosed in 23.3% (*n* = 126). The mean time after surgery for both groups combined was 6.1 ± 2.2 years and the mean duration of follow-up was 3.4 ± 2.3 years (*n* = 564).

No significant differences between IFU and LTFU patients were observed in terms of age, preoperative BMI, prevalence and therapy for diabetes mellitus, preoperative HbA1c, or arterial hypertension at baseline (Table 1).

Men were more likely to be LTFU, while patients who underwent conversion from an SG to an RYGB were significantly more likely to be IFU.

LTFU patients that could not be reached had their surgery longer ago. No other significant differences were observed between the subgroups (Supplemental Table S1). Comparing qLTFU patients based on the duration of follow-up of <2 years vs. ≥2 years (*n* = 114 vs. *n* = 105), there were no differences in age, preoperative BMI, prevalence and therapy of diabetes mellitus, or arterial hypertension and preoperative HbA1c (Supplemental Table S2). In the group of LTFU patients who took part in follow-up for at least two years there were significantly more women. The surgery of patients who were in follow-up for at least two years took place a mean of 7.2 years previously, whereas for patients who were in follow-up for a shorter time, the operation was only a mean of 6.4 years previously.

### 3.2. Body Weight

**Figure 2:**
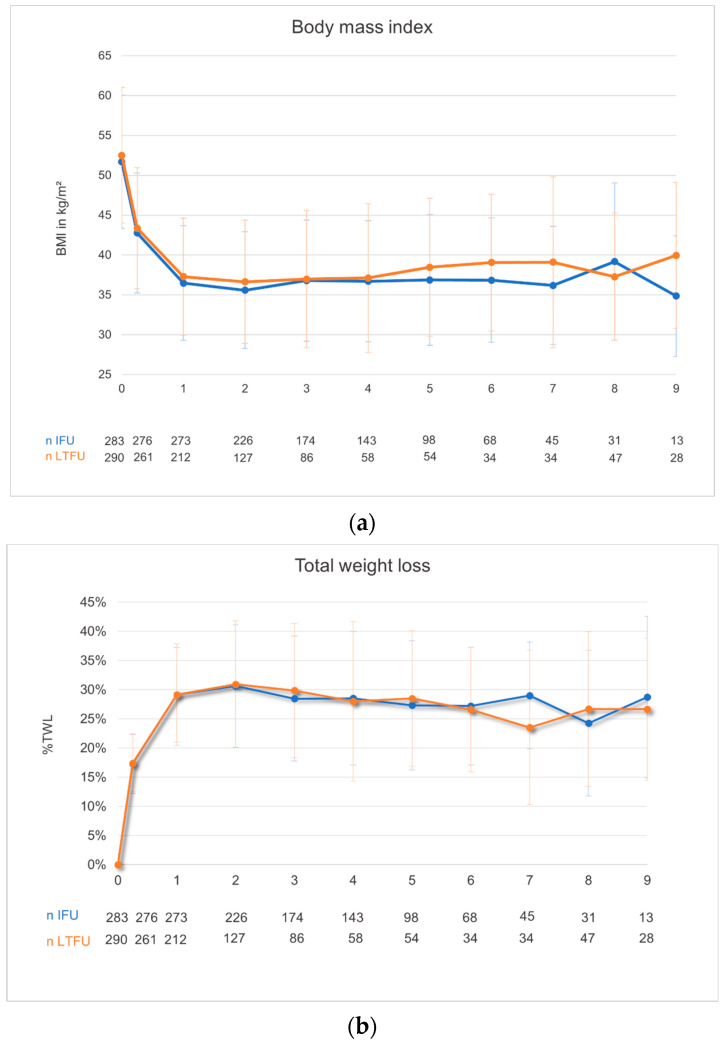
(**a**) Body mass index, BMI = body mass index; IFU = in follow-up; kg = kilogram; LTFU = lost to follow-up; m^2^ = square meter; n = number. (**b**) % Total weight loss, IFU = in follow-up; LTFU = lost to follow-up; n = number; TWL = total weight loss.

Postoperative body weight loss in IFU and LTFU patients is shown in Figure 2a,b. Preoperative BMIs were similar (51.7 vs. 52.5 kg/m^2^). Substantial weight loss was observed in both groups with its nadir during the second year after surgery (BMI 35.6 vs. 36.4 kg/m^2^, *p* = 0.28; % total weight loss (%TWL) 30.6% vs. 30.9%, *p* = 0.75). After a slight body weight regain two to five years after surgery, weight stabilisation was observed. No significant differences were found among the groups at any time point.

Subgroup analysis showed that %TWL of qLTFU patients with a follow-up duration < 2 years was significantly lower compared to qLTFU patients with a follow-up duration ≥ 2 years (%TWL 23.8% vs. 28.4%, *p* = 0.013) (Table 2).

### 3.3. Changes in Type 2 Diabetes and Hypertension

**Figure 3:**
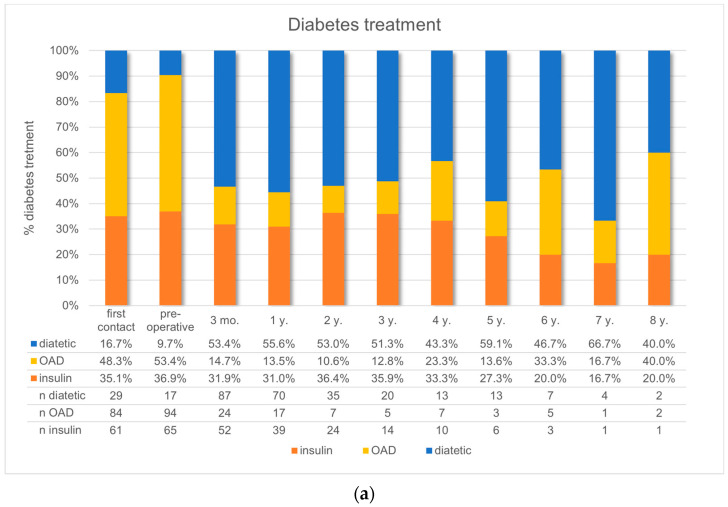

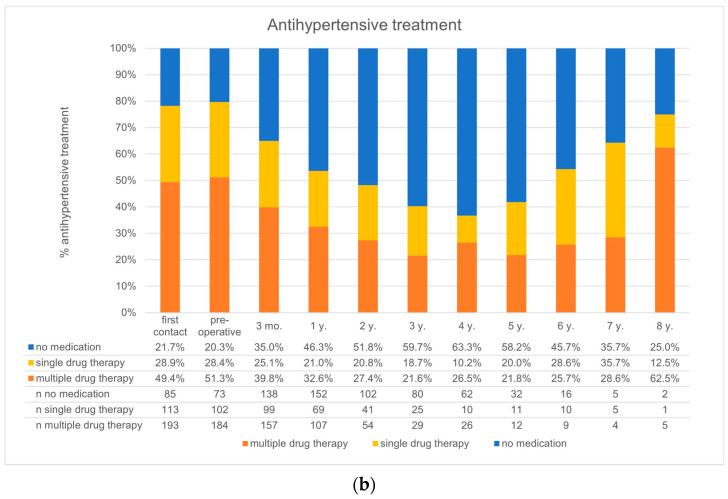
(**a**) Diabetes treatment in the whole study population (LTFU and IFU); mo. = months; *n* = number; NIAD = non-insulin antidiabetic treatment; y. = year. (**b**) Antihypertensive treatment in the whole study population (LTFU and IFU); mo. = months; *n* = number; y. = year.

Figure 3a shows the glucose-lowering treatment of all patients with available follow-up records with type 2 diabetes mellitus prior and after surgery. From the first visit to the appointment just prior surgery, there was an increase in type 2 diabetes diagnoses and an increase in the use of glucose-lowering medication. Three months postoperatively, there was a marked decrease in the use of non-insulin treatments from 53.4% to 14.7%. This effect persisted without a significant increase until year 8. The rate of insulin treatment decreased from 36.9% preoperatively to a nadir of 26.4% in year three postoperatively.

Figure 3b demonstrates the need for antihypertensive treatment prior and after surgery in all patients. The proportion of patients off antihypertensives increased from 20.3% preoperatively to a maximum of 63.3% within four years, while the use of both single and combination therapies decreased.

At the time of the telephone interview, which was in mean seven years after surgery, 76.7% of LTFU patients reported a reduction in their glucose-lowering medication and 63.9% in their antihypertensive medication (Table 3). Patients with RYGB had a greater reduction in glucose-lowering medication compared to patients with SG (Table 3). Patients who experienced the greatest improvements in hypertension were younger.

### 3.4. Structured Interview in LTFU Patients

**Figure 4:**
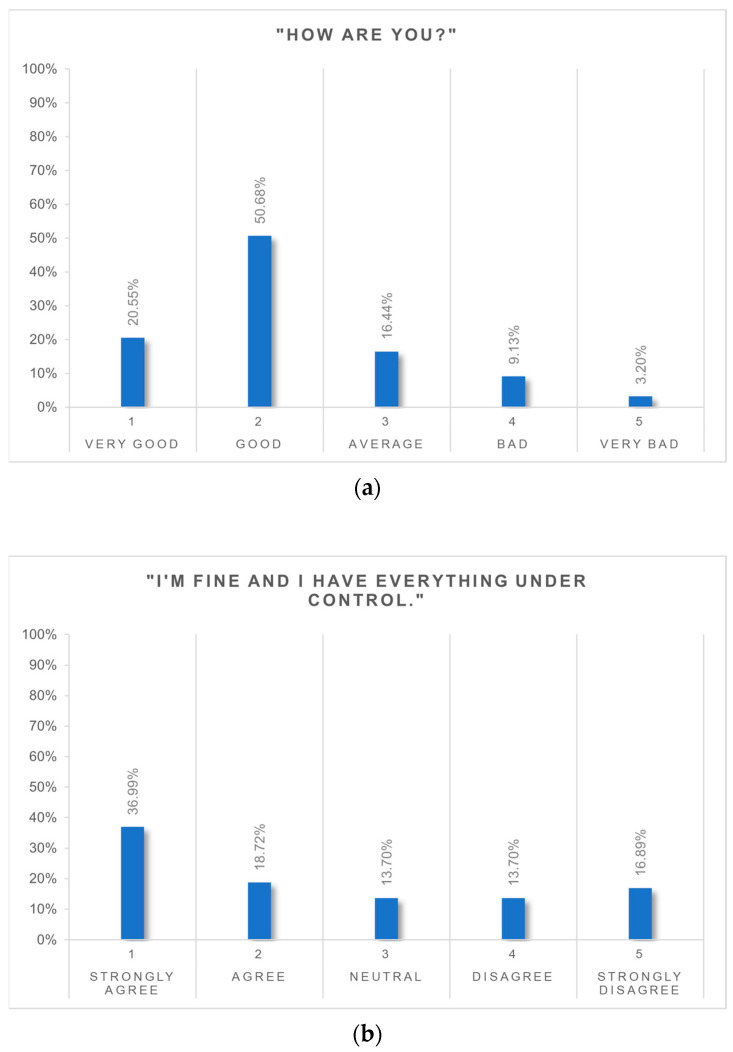
(**a**) “How are you?”. (**b**) “I’m fine and I have everything under control”.

Most qLTFU patients reported that they were feeling very well or well (71.2%) (Figure 4a). The statement ‘I am doing well and have everything under control’ applied to 55.3% of patients (Figure 4b), while 30.6% of patients reported that this statement did not apply to them.

A total of 180 patients (82.2%) reported that they made the right choice to undergo bariatric surgery; eight patients (3.7%) were uncertain, and 31 patients (14.1%) regretted having had surgery. Male patients were significantly more likely to be satisfied with their decision to undergo surgery (91.8% vs. 77.4%; p_c_ = 0.048). Patients who had a conversion from SG to RYGB answered the question “how are you?” with a significantly worse result (*p* = 0.021). They reported significantly more fear of regaining weight, than other patients (Table 4). The older the patients, the less satisfied they were with their outcomes (τ = 0.1; *p* = 0.033). A total of 128 patients reported no problems with eating since surgery (58.4%), while 91 patients (41.6%) reported having problems with eating. Overall, women reported problems significantly more often than men (48.0% vs. 28.8%; p_c_ = 0.006) (Table 4).

A diagnosis of depression was established in 18.3% of LTFU patients prior to surgery. Sixty-two patients (28.3%) confirmed experiencing psychological difficulties such as depression postoperatively. Of the 62 patients with psychological issues, 40 (64.5%) reported to receive professional care. Additionally, 34.3% of RYGB patients and 37.5% of patients who had conversion surgery reported psychological issues, as compared to 16.2% of SG patients (p_c_ = 0.014). A substantial portion of patients (151 = 69.1%) reported being afraid of weight gain, with women significantly more likely than men to express such concerns (74.7% vs. 57.5%, *p* = 0.011).

### 3.5. Reasons for Being LTFU

**Figure 5:**
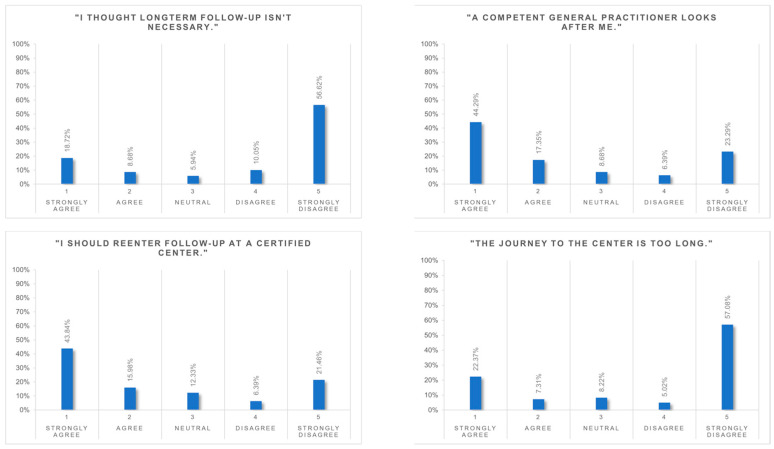

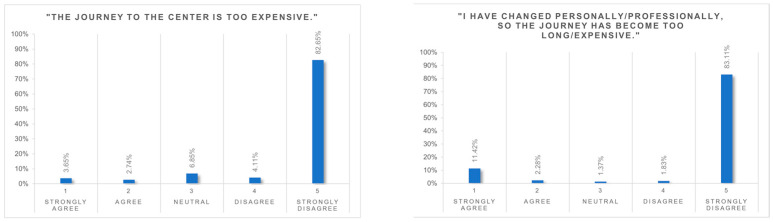
Reasons for being lost to follow-up (LTFU).

Figure 5 shows the given reasons for being LTFU. It is shown that 66.6% of patients were aware of the necessity of follow-up and stated that they should visit the obesity centre. Also, 61.6% reported having competent primary care physicians.

Travel issues were mentioned in 29.7% (long distance) and 6.4% (costs) of patients as a reason for being LTFU. Patients were also given the opportunity to mention their own reasons for being LTFU (Supplemental Table S3). The five most commonly mentioned reasons were health reasons (*n* = 19), dissatisfaction with treatment (*n* = 18), lack of time (*n* = 14), reasons related to work (*n* = 10) and difficulties with making an appointment (*n* = 10).

### 3.6. Supplementation of Micronutrients

Of the total, 77.6% of patients reported taking micronutrient supplements regularly with 64.9% of patients after SG, 75.0% after conversion surgery and 84.7% after RYGB (p_c_ = 0.0052). There were no significant differences in people taking supplements with regard to age, sex and comorbidities.

**Figure 6:**
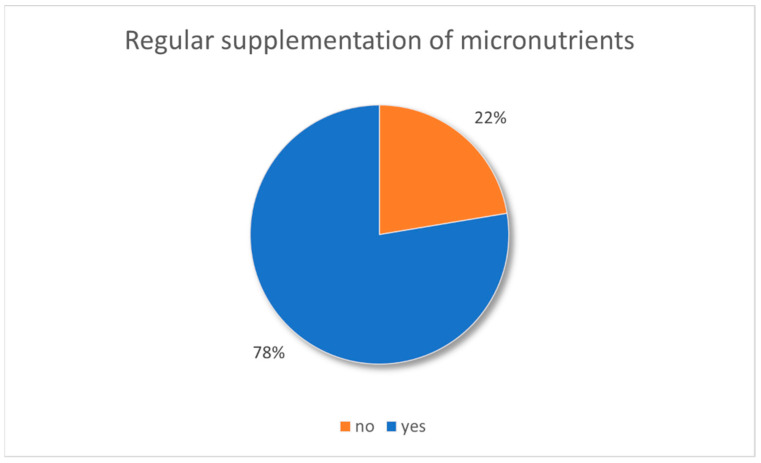

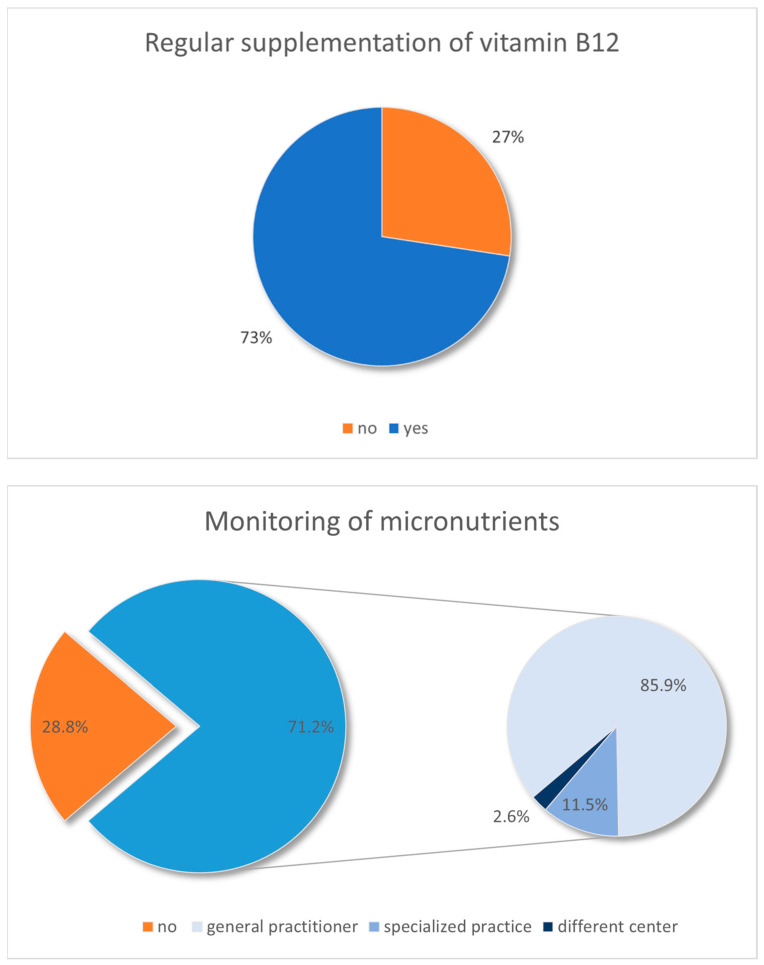
Supplementation and monitoring of micronutrients in LTFU patients.

Moreover, 72.6% of patients reported receiving vitamin B12 regularly. RYGB patients received vitamin B12 more regularly than patients after conversion or SG (80.3% vs. 62.5% vs. 59.5%; p_c_ = 0.0048). Also, 71.2% of patients reported that their vitamin status was regularly monitored, with no differences among the surgical groups. Patients who reported regular monitoring of their micronutrient status stated that this was performed by primary care physicians (85.9%), by a specialist practice (11.5%), or by another centre (2.6%) (Figure 6).

### 3.7. Demographics of Deceased Patients

Postoperatively, 16 patients died. One patient died within 30 days postoperatively due to an abdominal wall abscess causing septic multiorgan failure following surgery. The other deaths were not surgery related.

Deceased patients were older (53.1 ± 12.1 years), more obese (54.0 ± 10.0 kg/m^2^) and had more comorbidities with preoperative type 2 diabetes in 81.3% (*n* = 13) of them and arterial hypertension in 93.8% (*n* = 15).

The average time between surgery and death was 3.8 ± 2.7 years. The patients had been in follow-up for an average of 1.8 ± 2.2 years (*n* = 16). The longest follow-up duration was 8.2 years. In all, 50.0% of the deceased patients were formally LTFU.

## 4. Discussion

This study examined the postoperative patient-related long-term outcomes and follow-up adherence of patients with obesity undergoing bariatric surgery at a tertiary certified centre.

With an average age of 44.1 years, initial BMI of 52.1 kg/m^2^, an established diagnosis of type 2 diabetes in 33% and hypertension in 74% of patients, our data confirm that patients seeking bariatric surgery in Germany tend to be older, have a higher BMI and have a higher prevalence of obesity-associated complications when compared to international data in the IFSO registry [20,24].

Our follow-up rate of 90.3% after one year, 44.6% after five and 15.0% after ten years confirms previous studies demonstrating that a significant portion of patients drop out of regular routine follow-up [12,13]. In contrast, follow-up rates have often exceeded 90% in prospective studies even after ≥10 years [1], which does not reflect the real-world care [12,13] but may be explained by ‘surveillance bias’ [25].

Reproducible predictors for being LTFU after bariatric surgery are not well established [26]. In this study, men were significantly more often LTFU, which is consistent with the literature [13,27,28]. This could be explained by an overall superior health-conscious behaviour in women [29]. Another significant parameter was the overall follow-up duration, which was in line with the literature [13]. Age, preoperative BMI, and comorbidities had no influence on the follow-up status.

To compare outcomes from IFU and LTFU patients, PROs from LTFU patients were prospectively collected via structured telephone interviews. In this context, this study is one of the largest of its kind [30,31,32,33]. The validity of PROs regarding body weight and comorbidity data in patients with severe obesity has been demonstrated previously [34,35,36].

Patients who were not available despite several attempts had their surgery and last visit to the centre significantly longer ago than people who were. A possible explanation might be that they had more likely moved and/or changed their contact details. Aside from the longer time since surgery, no other significant differences were observed between the LTFU subgroups.

Overall, 2.7% of patients had died over a ten-year period, which is similar to previous findings regarding mortality after surgery with a mortality rate of 6.2% after twelve [2] and of 4.2% after ten years [37]. The deceased patients were older, sicker, and had higher BMIs at the time of surgery. Half of the deceased patients had fulfilled the definition of LTFU. The perioperative 30-day mortality rate in this study was 0.2%, which is comparable to the literature [38].

Even though our data show that discontinuing follow-up did not negatively affect postoperative outcomes in terms of weight loss and reported improvement in comorbidities such as type 2 diabetes and hypertension, the duration of follow-up had an effect on weight-loss outcomes.

In line with the literature, %TWL was 30% two years after surgery and stabilised at about 27% after five years [1,2,39,40], with no differences between IFU and LTFU cohorts. In a meta-analysis, a significantly higher weight loss was observed in the IFU group during short-term observation of up to three years postoperatively. After three years, however, no difference in weight loss regarding follow-up status was observed [17].

Recent studies suggest that attending standardised follow-ups during the first three postoperative years affects weight loss in the long-term, while participation in follow-up beyond this period is not associated with %TWL [17]. In line with this, participation in follow-up for at least two years after surgery was associated with higher %TWLs comparable to what has previously been shown [1,2,39,40]. These data suggest that the first postoperative years are a particularly important phase and that intensive follow-up care and training at the respective obesity centre in the first years could pave the way for the consistent success of surgery even after LTFU.

### 4.1. Postoperative Satisfaction and Mental Well-Being

The majority of LTFU patients reported that they were feeling “well” or “very well” with regards to their wellbeing. Consistent with the literature, over 80% stated that they were satisfied with their decision to undergo surgery [31,32,41]. Patients’ satisfaction in this study was higher in men, wwhile sex-related differences remained inconsistent in this context [42]. Fear of regaining weight was significantly higher in women, although there were no relevant sex-related differences in weight regain after bariatric surgery in a matched cohort study [43]. Postoperative weight loss and improvement in comorbidities are the main reasons for women to undergo bariatric surgery [44]. Thereby, expectations on the amount of postoperatively achieved weight loss are more likely to be unrealistic in female patients compared to their male counterparts [45]. Not achieving the expected goals may lead to more dissatisfaction and, conversely, could explain the greater fear of regaining weight. This fear in women might also be fuelled by a lower sense of self-efficacy and a more pronounced suffering from the stigma of obesity.

While most qLTFU patients reported feeling well, only half of the patients agreed that they were “doing well and have everything under control”, while about a third disagreed. This could show a noticeable lack of professional support for a considerable amount of LTFU patients.

Preoperatively, a depression diagnosis among qLTFU patients was established in 18.3% which is consistent to previous reports showing a high rate of psychological disorders among patients with obesity [46]. Nearly 30% of LTFU patients reported that they had experienced psychological problems postoperatively. A recent study showed that 10.2% of patients undergoing bariatric surgery without a previous history of depression had positive depression screening before surgery, suggesting a relevant rate of undiagnosed depressive disorders [47]. Overall, the current literature suggests an initial improvement in depression after surgery, followed by a deterioration in mood [46,48,49].

### 4.2. Reported Reasons for Being LTFU

Travel costs were reported to not be a factor contributing to LTFU in over 85% of patients. Nevertheless, consistent with the literature, travel duration was the most frequently mentioned reason for being LTFU [31,32].

Even though the necessity of postoperative lifelong follow-up had been emphasised before surgery, 25% of patients reported that they thought it was unnecessary, which is in range (9–29.5%) of what has been published previously [31,32]. An explanation could be that these patients are doing well and do not see an additional benefit from routine centre-based follow-up. This observation is supported by the patients’ high agreement (60%) to the statement: “I have competent primary care physicians who take care of me.”

Patients also had the opportunity to mention their own reasons for dropout. Health reasons as well as lack of time and work-related reasons have been described earlier as main reasons [31,32,33]. Having regained weight was mentioned by nine patients in our cohort, thus also comparable to previous interviews [31,32,33]. Additionally, eighteen patients expressed dissatisfaction with the treatment (doctor’s consultations not supportive enough, consultation time not sufficient relative to travel time). In the literature, 7% of patients interviewed by Luca et al. also cited a loss of trust in the treating physicians [30]. Further reasons for LTFU were only mentioned by a few patients.

### 4.3. Status of Care in LTFU Patients

Over 77% of LTFU patients stated that they regularly supplement micronutrients, and slightly fewer (72.6%) that they regularly receive vitamin B12. Almost as many patients stated that their micronutrient status was regularly monitored. As this was mostly performed by primary care physicians, while only a minority stated that they were being treated at a specialised practice or another obesity centre, it is questionable whether the recommended laboratory parameters are routinely controlled as these are not reimbursed and the needed expertise might be missing [7].

The reported noncompliance rate regarding micronutrient supplementation is within the range (17.2% and 22.3%) of what has previously been published [50,51,52], with patients stopping taking their supplements over time [53]. In this context, it has been shown that IFU patients are more likely to take necessary supplements and have fewer deficiencies [54,55]. Thus, especially combined with no adequate follow-up there seems to be a significant number of LTFU patients who are at evident risk of developing deficiencies due to a lack of supplementation and control.

### 4.4. Strengths and Limitations

A strength of this study is its prospective design and a relatively large number of patients, allowing subgroup analyses. The structured interview was conducted with 219 out of 290 LTFU patients, not only making it the largest survey of patients after SG or RYGB regarding health status and reasons for dropping out of follow-up to date [30,31,32,33], but also including the vast majority of patients of interest. Prior to the interview, a common interview strategy was agreed upon. The interview was standardised and conducted mainly with closed questions. However, 17.9% of patients were still not available for the interview—baseline criteria suggested no systematic difference between patients available and those who were not.

The study also has limitations. Firstly, it is a single-centre study. Further, the results of the prospective telephone interview are based on PROs, which were not objectively validated in this study. Especially for questions covering a longer period, a recall bias is possible. With this design, it was also not possible to compare micronutrient levels between patients LTFU and IFU.

The definition of “LTFU” varies widely in the literature [16,26,31,32,56] thus limiting the comparability of studies. Some authors classified patients as LTFU if they missed just one appointment [16,56], or if participation was irregular [32], or if appointments for at least six months were missed [31]. It should be considered that most of the FU studies cited here have a very short follow up duration of 12 months to a maximum of five years. The longer the follow-up duration, the more likely it is for appointments to be missed. To account for possible appointment shifts, an interval of 1.5 years was chosen in this work. Thus, the criteria chosen here to be classified as LTFU are not as strict as in most other studies.

## 5. Conclusions

Reproducible predictors for being LTFU after bariatric surgery apart from being male were not found. Though not frequently mentioned, travel issues were the most common reasons for LTFU. Health reasons, dissatisfaction with the treatment and lack of time were also often mentioned. LTFU patients showed high satisfaction and reported comparable outcomes to IFU patients, but %TWL was lower in patients who left follow-up early. Even in cases of comparable weight loss, lifelong follow-up of bariatric surgery patients is most likely essential due to the risk of nutritional deficiencies, as a substantial proportion of LTFU patients denied regular supplementation and 29% reported not having checked their micronutrient levels regularly. As many LTFU patients see their general practitioner regularly, it is important to involve general practitioners in follow-up care as long as professional training and reimbursement for appropriate laboratory tests are provided after an initial follow-up period at a specialist centre.

## Figures and Tables

**Table 1:** Baseline characteristics of IFU and LTFU patients.

	IFU	LTFU	*p*-Value
**Patients** (*n* = 573)	*n* = 283 (%)	*n* = 290 (%)	
**Sex (/♂)** (*n* = 406/167)	214/69 (75.6/24.4)	192/98 (66.2/33.8)	p_c_ = 0.013
**Age** (years) at surgery	44.3 ± 10.7	43.8 ± 11.6	p_U_ = 0.66
**Preoperative BMI** (kg/m^2^)	51.7 ± 8.3	52.5 ± 8.5	p_U_ = 0.37
**Diabetes mellitus type 2** (*n* = 531)	93/273 (34.1)	83/258 (32.2)	p_c_ = 0.64
**dietary treatment (*n* = 17)**	7 (7.5)	10 (12.1)	p_c_ = 0.60
**non-insulin antidiabetic treatment (*n* = 94)**	51 (54.8)	43 (51.8)	
**insulin treatment (*n* = 65)**	35 (37.6)	30 (36.1)	
**HbA1c preoperative (%)**	7.4 (6.0; 9.2) ^1^	7.4 (6.1; 8.9) ^2^	p_t_ = 0.89
**duration of diabetes (years prior surgery)**	10.7 (2.9; 39.8) ^3^	12.7 (3.9; 41.7) ^4^	p_t_ = 0.44
**Hypertension** (*n* = 531)	204/273 (74.7)	191/258 (74.0)	p_c_ = 0.85
**no medication (*n* = 67)**	34 (18.4)	33 (19.6)	p_c_ = 0.47
**single drug antihypertensive therapy (*n* = 102)**	49 (26.5)	53 (31.6)	
**multiple drug antihypertensive therapy (*n* = 184)**	102 (55.1)	82 (48.8)	
**Type of surgery**			
**SG (*n* = 185)**	83 (29.3)	102 (35.2)	p_c_ = 0.0051
**RYGB (*n* = 353)**	174 (61.5)	179 (61.7)	
**Conversion SG** **→** **RYGB (*n* = 35)**	26 (9.2)	9 (3.1)	
**Time operation—last follow-up** (years)	4.6 ± 2.2 ^5^	2.2 ± 1.8	p_U_ < 0.0001
**Time operation—cutoff date ^6^** (years)	5.2 ± 2.1	7.0 ± 2.0	p_U_ < 0.0001

^1^ *n* = 69; ^2^ *n* = 49; ^3^ *n* = 66; ^4^ *n* = 60; ^5^ *n* = 274; ^6^ 1 July 2019. Data are mean ± SD or number (%); BMI = body mass index; IFU = in follow-up; kg = kilogram; LTFU = lost to follow-up; m^2^ = square meter; *n* = number; *p* = *p*-value; RYGB = Roux-en-Y gastric bypass; SG = sleeve gastrectomy.

**Table 2:** %TWL depending on follow-up duration.

	Follow-up Duration	*n*	Mean Value	p_U_
**%TWL**	<2 years	106	23.8 ± 14.7	0.013
	≥2 years	94	28.4 ± 11.3	

*n* = number; *p* = *p*-value; TWL = total weight loss.

**Table 3:** Change in medication for concomitant diseases reported in the qLTFU.

		Antidiabetic Medication			Antihypertensive Medication		
		*n*	%	p_mp_	*n*	%	p
**Total**	Reduced	56	76.7		83	63.9	
	Unchanged	16	21.9		33	25.4	
	Increased	1	1.4		14	10.8	
**Sex** (♀/♂)	Reduced	32/24	82.1/70.6	0.14	54/29	67.5/58.0	p_c_ = 0.055
	Unchanged	6/10	15.4/29.4		15/18	18.8/36.0	
	Increased	1/0	2.6/0.0		11/3	13.8/6.0	
**RYGB**	Reduced	39	88.6	0.007	59	71.1	p_mp_ = 0.100
	Unchanged	5	11.4		17	20.5	
	Increased	0	0.0		7	8.4	
**SG**	Reduced	14	53.9		22	48.9	
	Unchanged	11	42.3		16	35.6	
	Increased	1	3.9		7	15.6	
**Conversion**	Reduced	3	100.0		2	100.0	

*n* = number; *p* = *p*-value; RYGB = Roux-en-Y-gastric bypass; SG = sleeve gastrectomy.

**Table 4:** Problems with eating and fear of weight regain.

	Problems with Eating			Fear of Weight Regain		
	***n***	**%**	**p_c_**	***n***	**%**	**p_c_**
**Total** (*n* = 219)	91	41.6		151	69.0	
**Female** (*n* = 146)	70	48.0	0.006	109	74.7	0.011
**Male** (*n* = 73)	21	28.8		42	57.5	
**SG** (*n* = 137)	54	39.4	0.14	101	73.7	0.032
**RYGB** (*n* = 74)	31	41.9		43	58.1	
**Conversion** (*n* = 8)	6	75.0		7	87.5	

*n* = number; *p* = *p*-value; RYGB = Roux-en-Y-gastric bypass; SG = sleeve gastrectomy.

## Data Availability

The raw data supporting the conclusions of this article will be made available by the authors on request.

## Supplementary Materials

The following supporting information can be downloaded at: https://www.mdpi.com/article/10.3390/nu16162710/s1, Table S1: Baseline characteristics of LTFU subgroups; Table S2: Baseline characteristics of LTFU patients depending on follow-up duration; Table S3: Additionally mentioned reasons for being lost to follow-up; Table S4: Supplementation of micronutrients depending on time in follow-up; Supplementary Files S1: structure of the standardised interview; Supplementary Files S2: dataset.
