# Supplementary material for: Characteristics of Patients Lost to Follow-up after Bariatric Surgery
Source: Nutrients. 2024 Aug 15;16(16):2710. doi: 10.3390/nu16162710 (PMC11357598; doi:10.3390/nu16162710)
Supplement: Supplementary file 1 [file nutrients-16-02710-s001.zip › nutrients-3150364-supplementary.pdf]

Supplementary Table S1: Baseline characteristics of LTFU subgroups

	qLTFU	Not interested	Not reached	p-value
Patients (n=290)	219 (75.5)	19 (6.6)	52 (17.9)	
Sex (♀/♂) (n=192/98)	146/73 (66.7/33.3)	15/4 (79.0/21.1)	31/21 (59.6/40.4)	p_c=0.29
Age (years)	43.9 ± 11.7	42.8 ± 10.5	44.1 ± 11.8	p_{KW}=0.83
BMI (kg/m²)	52.6 ± 8.5	52.6 ± 9.2	52.2 ± 8.4	p_{KW}=0.99
Diabetes mellitus type II (n=268)	61/195 (31.3)	4/16 (25.0)	18/47 (38.3)	p_c=0.54
- dietary treatment (n=10)	9 (14.8)	0 (0.0)	1 (5.6)	
- non-insulin antidiabetic medication (n=43)	29 (47.5)	3 (75.0)	11 (61.1)	
- insulin treatment (n=30)	23 (37.7)	1 (25.0)	6 (33.3)	p_{mp}=0.76
- HbA_{1c} preoperative (%)	7.5 (6.1; 9.1)[1]	6.9 (5.9; 8.1)[2]	7.1 (6.0; 8.4)[3]	p_a=0.65
- Duration of diabetes (years)	6.4 (1.7; 14.0)[4]	14.0[5]	6.1 (1.8; 12.9)[6]	p_a=0.89
Hypertension (n=258)	142/195 (72.8)	9/16 (56.3)	40/47 (85.1)	p_c=0.053
- no medication (n=82)	26 (20.8)	1 (12.5)	6 (17.1)	
- single drug antihypertensive therapy (n=53)	39 (31.2)	3 (37.5)	11 (31.4)	
- multiple drug antihypertensive therapy (n=23)	60 (48.0)	4 (50.0)	18 (51.4)	p_c=0.96
Type of operation				
- SG	74 (33.8)	8 (42.1)	20 (38.5)	
- RYGB	137 (62.6)	11 (57.9)	31 (59.6)	
- Conversion SG → RYGB	8 (3.7)	0 (0.0)	1 (1.9)	N/A
Time operation – last follow-up (years)	2.3 ± 1.8	1.5 ± 1.3	2.3 ± 2.2	p_{KW}=0.098
Time operation – cutoff date[7] (years)	6.8 ± 2.0	6.3 ± 2.1	7.8 ± 1.6	p_{KW}=0.0042

[1]n=35; [2]n=4; [3]n=10; [4]n=48; [5]n=1; [6]n=11; [7]01.07.2019

Data are mean ±SD or number (%); BMI = body mass index; IFU = in follow-up; kg = kilogram; LTFU = lost to follow-up; m² = square meter; n = number; p = p-value; qLTFU = questioned lost to follow-up; RYGB = Roux-en-Y gastric bypass; SG = sleeve gastrectomy

Supplementary Table S2: Baseline characteristics of LTFU patients depending on follow-up duration

		Follow-up duration		p-value
	qLTFU	<2 years	≥ 2 years	
Patients	n=219 (%)	n=114 (%)	n=105 (%)	
Sex (♀/♂)	146/73 (66.7/33.3)	69/45 (60.5/39.5)	77/28 (73.3/26.7)	p_c=0.044
Age (years)	43.9 ± 11.7	44.9 ± 11.5	42.8 ± 11.9	p_U=0.23
BMI (kg/m²)	52.6 ± 8.6	52.1 ± 9.0	53.1 ± 8.0	p_U=0.21
Diabetes mellitus type II	61/195 (31.3)	35/100 (35.0)	26/95 (27.4)	p_c=0.25
- dietary treatment	9 (14.8)	3 (8.6)	6 (23.1)	
- non-insulin antidiabetic medication	29 (47.5)	20 (57.1)	9 (34.6)	
- insulin treatment	23 (37.7)	12 (34.3)	11 (42.3)	p_c=0.13
HbA_{1c} preoperative (%)	7.5 (6.1; 9.1)[1]	7.1 (5.8; 7.3)[2]	7.9 (6.5; 9.6)[3]	p_a=0.14
Duration of diabetes	6.4 (1.7; 14.0)[4]	5.6 (1.2; 13.4)[5]	7.4 (2.5; 14.7)[6]	p_a=0.33
Hypertension	142/195 (72.8)	72/100 (72.0)	70/95 (73.7)	p_c=0.79
- no medication	26 (20.8)	10 (16.7)	16 (24.6)	
- single drug antihpyertensive therapy	39 (31.2)	20 (33.3)	19 (29.2)	
- multiple drug antihypertenisive therapy	60 (48.9)	30 (50.0)	30 (46.25)	p_c=0.54
Type of operation				
- SG	74 (33.8)	45 (39.5)	29 (27.6)	
- RYGB	137 (62.6)	67 (58.8)	70 (66.7)	
- Conversion SG → RYGB	8 (3.7)	2 (1.8)	6 (5.7)	p_c=0.072
Time operation – last follow-up (years)	2.3 ± 1.8	1.0 ± 0.6	3.7 ± 1.5	p_U<0.0001
Time operation – cutoff date[7] (years)	6.8 ± 2.0	6.5 ± 2.2	7.2 ± 1.8	p_U=0.0082

[1]n=35; [2]n=20; [3]n=15; [4]n=48; [5]n=25; [6]n=23; [7]01.07.2019

Data are mean ±SD or number (%); BMI = body mass index; IFU = in follow-up; kg = kilogram; LTFU = lost to follow-up; m² = square meter; n = number; p = p-value; qLTFU = questioned lost to follow-up; RYGB = Roux-en-Y gastric bypass; SG = sleeve gastrectomy

Supplementary Table S3: Additionally mentioned reasons for being lost to follow-up

Reason	Number of mentions
Health-related reasons	19
Unsatisfied with treatment	18
No time	14
Professional reasons	10
Difficulties with making an appointment	10
Feeling of shame	9
Forgot appointment	6
Waiting time	5
Changed doctor	4
Family-related reasons	3
No need	2
Pregnancy	2
Lack of motivation	2
Staying abroad	1

Supplementary Table S4: Supplementation of micronutrients depending on time in follow-up

	< 2 years follow-up duration		≥ 2 years follow-up duration		
	n	%	n	%	p_c
Regular intake of vitamins and trace elements	89/114	78.1	81/105	77.1	0.87
Regular intake of vitamin B12	80/114	70.2	79/105	75.2	0.40
Monitoring of vitamins and trace elements	86/114	75.4	70/105	66.7	0.15

Supplementary Files S1:

Structure of the standardized interview:

My name is ... from the Obesity Centre of the University Hospital of Wuerzburg.

You underwent bariatric surgery at the Wuerzburg Obesity Centre on xx.xx.20xx. When reviewing your records, we realised that you have not been in our follow-up programme for xx months. We would like to conduct a short, structured telephone interview, which will take approx. 5-10 minutes of your time, to find out about your current state of health and the reasons why you are no longer attending our follow-up care and offer you an appointment at our centre if you wish. Our aim is to improve the care of bariatric surgery patients and, above all, to offer you our help.

It is very important to us that this call should not be a warning or a criticism for you. Rather, we want you to be well looked after.

Your data will be analysed anonymously for scientific purposes. For certain analyses, we need a few more basic details about you and your operation. However, none of the answers can be attributed to you personally or to your patient data.

We are conducting this study in order to further improve our aftercare services in the future and make them more easily accessible for our patients.

"Do you agree with this?" (yes/no)

<u>Basic data</u>

1. date of birth

2. sex

3. height

4. date of surgery

5. surgical procedure

6. preoperative weight

7. last follow-up at the Würzburg Obesity Centre

<u>Structured telephone interview</u>

1. "How are you doing?" *(1 = "very good" to 5 = "very bad")*

2. "What were the reasons why you did not come to our centre for follow-up care?" *(1 = "applies" to 5 = "does not apply at all"):*

a. "I didn't think it was necessary."

b. "I'm fine and I have everything under control."

c. "I have a competent doctor in private practice who looks after me."

d. "I should actually go back to the centre."

e. "The journey is too long for me."

f. "The journey is too expensive for me."

g. "I have changed personally/professionally, so the journey has become too long/too expensive."

h. "I am being treated at another obesity centre."

i. Is there another reason? *(free text)*

3. "How did the weight develop after the operation?"

   a. "What was the lowest weight after the operation?"

   b. "What is your current weight?"

4. "Have you had any problems with eating since the operation? For example, do you vomit more often?" *(yes/no)*

5. "Are you afraid of gaining weight again?" *(yes/no)*

6. "How have your concomitant illnesses developed?" *(improved/worsened/stayed the same)*

   a. Diabetes mellitus type 2

   b. Arterial hypertension

   c. Joint problems

   d. Other

7. "Has your medication changed?" *(increased/reduced/stayed the same)*

   a. Antidiabetics/insulin

   b. Antihypertensives

   c. Other medications

8. "Have any new illnesses arisen since the operation?" *(yes/no)*

   a. "If yes, which illnesses have been added?"

9. "Were you mentally unwell after the operation? For example, did you suffer from depression? *(yes/no)*

   a. "If yes, did you receive professional counselling from a psychologist?" *(yes/no)*

10. "Do you regularly take vitamins and trace elements?" *(yes/no)*

   a. "Do you regularly take vitamin B12?" *(yes/no)*

   b. "Do you have your vitamin status checked?" *(yes/no)*

   c. "If yes, who checks it?" *(General practitioner/specialist practice/other centre)*

11. "Have you undergone abdominal surgery again after the bariatric surgery? *(yes/no)*

   a. "If yes, which one?"

   b. "If yes, was this operation an emergency operation?" *(yes/no)*

12. "Have you had orthopaedic surgery since then?" *(yes/no)*

   a. "If yes, which operation was performed?"

   b. "If no, is surgery currently indicated?" *(yes/no)*

13. "Have you undergone plastic surgery?" *(yes/no)*

   a. "If yes, which body regions were operated on?"

   b. "If yes, are you satisfied with the result?" *(yes/no)*

   c. "If no, was the performance of such an operation an issue?" *(yes/no)*

14. "Have you had another operation since the operation due to your obesity or the bariatric surgery we performed?" *(yes/no)*

   a. "If yes, which operation was performed and when?"

15. "I would have the bariatric surgery performed again?" *(yes/no)*

16. "May we contact you to offer you a follow-up appointment?" *(yes/no)*

17 "Do you have any questions or comments?"

18. for patients after gastric sleeve surgery:

   a. "Do you suffer from heartburn?" *(yes/no)*

b. "If yes, how often?" *(very often/frequently/occasionally/rarely/very rarely)*

c. "If yes, how severe?" *(1 = "not bad" to 5 = "very bad")*

d. "If yes, do you take medication for it?" *(yes/no)*
